# Supplementary material for: Effects of Water Restriction and Water Replenishment on the Content of Body Water with Bioelectrical Impedance among Young Adults in Baoding, China: A Randomized Controlled Trial (RCT)
Source: Nutrients. 2021 Feb 8;13(2):553. doi: 10.3390/nu13020553 (PMC7915432; doi:10.3390/nu13020553)
Supplement: Supplementary file 1 [file nutrients-13-00553-s001.pdf]

Table S1 The characteristics of participants

	Baseline test					Dehydration test							Rehydration test					
	WR group 1 (n=20)	WR group 2 (n=20)	WR group 3 (n=18)	NR group (n=18)	Total	WR group 1 (n=20)	WR group 2 (n=20)	WR group 3 (n=18)	NR group (n=18)	Total	*t*	*P*	WR group 1 (n=20)	WR group 2 (n=20)	WR group 3 (n=18)	NR group (n=18)	*F*	*P*
Age （year）	20.9±0.9	21.1±0.6	21.1±1.3	21.1±1.2	21.0±1.0	20.9±0.9	21.1±0.6	21.1±1.3	21.1±1.2	21.0±1.0	-	-	20.9±0.9	21.1±0.6	21.1±1.3	21.1±1.2	-	-
Height (cm)	168.3±8.1	167.1±7.8	163.5±7.9	165.9±9.0	166.3±8.2	168.3±8.1	167.1±7.8	163.5±7.9	165.9±9.0	166.3±8.2	-	-	168.3±8.1	167.1±7.8	163.5±7.9	165.9±9.0	-	-
Weight (kg)*	63.7±9.0	64.4±13.8	58.9±11.9	60.8±10.2	62.1±11.4	63.4±9.2	63.9±13.8	58.5±11.8	60.4±10.2	61.6±11.4	6.985	<0.001	63.9±9.2	64.0±13.8	58.5±11.7	60.2±10.1	1.102	0.354
BMI (kg/m^2)*	22.6±3.6	22.9±3.8	21.9±3.4	22.0±2.3	22.4±3.3	22.5±3.6	22.7±3.7	21.8±3.3	21.8±2.3	22.2±3.3	7.162	<0.001	22.7±3.7	22.8±3.7	21.8±3.3	21.8±2.3	0.544	0.654
Systolic pressure (mmHg)*	112±14	110±12	109±13	110±11	110±12	109±10	107±9	109±11	107±8	108±10	3.142	0.002	113±12	109±10	110±11	108±11	0.842	0.475
Diastolic pressure (mmHg)*	67±8	67±11	63±9	66±7	65±8	88±7	86±10	87±9	86±8	87±8	30.874	<0.001	67±8	64±8	64±9	65±8	0.334	0.800

Note: *, There were significant differences between the baseline test and dehydration test; Values are shown as the mean ± standard deviation (SD). Compare the baseline test and dehydration test, differences were found in weight, BMI, systolic pressure and diastolic pressure, respectively (t=6.985, p＜0.001; t=7.162, p＜0.001; t=3.142, p=0.002; t=30.874, p＜0.001).

Table S2 The temperature and humidity of study days

	Indoors		Outdoors	
	Temperature (℃)	Humidity (%)	Temperature (℃)	Humidity (%)
First study day	20.7	59	22.0	64
Second study day	20.5	57	20.4	55
Third study day	20.5	63	19.8	61

Table S3 The thirst, urine and plasma biomarkers of participants

	Baseline test					Dehydration test							Rehydration test					
	WR group 1 (n=20)	WR group 2 (n=20)	WR group 3 (n=18)	NR group (n=18)	Total (n=76)	WR group 1 (n=20)	WR group 2 (n=20)	WR group 3 (n=18)	NR group (n=18)	Total (n=76)	*t*	*P*	WR group 1 (n=20)	WR group 2 (n=20)	WR group 3 (n=18)	NR group (n=18)	*F*	*P*
Plasma Osmolality (mOsm/kg) *	293±5	293±7	294±5	293±4	293±5	297±6	298±4	296±5	296±4	297±5	-6.157	**<0.001**	292±4†	294±4	293±5	295±6	2.035	0.117
Urine Osmolality (mOsm/kg) *	840±261	829±170	786±205	830±234	822±217	1111±130	1079±114	1093±71	1092±78	1094±101	-11.221	**<0.001**	453±273$^{\#\dagger}$	888±352†	921±265†	1131±72	22.242	**<0.001**
USG	1.025±0.006	1.025±0.004	1.024±0.006	1.026±0.005	1.025±0.005	1.028±0.004	1.029±0.003	1.029±0.004	1.029±0.002	1.029±0.003	-5.019	**<0.001**	1.014±0.009$^{\dagger\#}$	1.025±0.008	1.026±0.007	1.029±0.004	17.966	**<0.001**
Hydration statuses (%)*																		
Dehydration	13（65.0）	12（60.0）	9（50.0）	10（55.6）		20（100.0）	20（100.0）	18（100.0）	18（100.0）				3（15.0）$^{\#\dagger}$	15（75.0）†	14（77.8）	18（100.0）		
Middle hydration	5（25.0）	8（40.0）	8（44.4）	5（27.8）		0（0.0）	0（0.0）	0（0.0）	0（0.0）		46.973	**<0.001**	4（20.0）	0（0.0）	2（11.1）	0（0.0）	43.172	**<0.001**
Optimal hydration	2（10.0）	0（0.0）	1（5.6）	3（16.7）		0（0.0）	0（0.0）	0（0.0）	0（0.0）				13（65.0）	5（25.0）	2（11.1）	0（0.0）		

Note: Values are shown as the mean ± standard deviation (SD), with the exception that percentages were shown as n (percentage); *, There were significant differences between the baseline and water

restriction of 36h; [#], there were significant differences between the four groups (WR group 1, WR group 2, WR group 3 and NR group) after water restriction of 36h; [†], there were significant differences between water restriction of 36h and water supplementation within the group. Compared with baseline test, the osmolality of plasma increased (t=-6.157, p<0.001) in dehydration test. The thirst, the osmolality of urine and USG increased (t=-6.758, p<0.001; t=-11.221, p<0.001; t=-5.019, p<0.001), significant differences were found in the hydration statuses (F=46.973, p<0.001). Compared with dehydration test, in rehydration test, there were significant decreases in WR group 1, WR group 2 and WR group 3 in thirst (t=8.661, p<0.001; t=6.099, p<0.001; t=2.605, p=0.018), no significant increase was found in NR group (t=-1.169, p=0.259); the osmolality of urine in WR group 1, WR group 2 and WR group 3 decreased (t=11.247, p<0.001; t=2.440, p=0.025; t=2.533, p=0.021), but there was a significant increase in NR group (t=-2.886, p=0.010); in hydration status, significant differences were found in WR group 1 and WR group 2 (^{2}=32.039, p<0.001; ^{2}=7.648, p=0.047), no significant differences were found in WR group 3 and NR group (^{2}=3.743, p=0.104; p=1.000). When compared the thirst during rehydration test, the improvement of thirst in WR group 1 was great than WR group 2 (t=-3.204, p=0.004), but no significant difference was found between WR group 2 and WR group 3 (t=-1.666, p=0.104); in the osmolality of urine, WR group 1 was lower than WR group 2 (t=-4.365, p<0.001), no significant difference was found between WR group 2 and WR group 3 (t=-0.324, p=0.747); in hydration statuses, WR group 1 was better than that in WR group 2 (^{2}=15.397, p< 0.001).

Table S4. Fluids from food, 24h urine volume, void number and urine osmolality on day 2.

	WR group 1 (n=20)	WR group 2 (n=20)	WR group 3 (n=18)	NR group (n=18)	Total (n=76)	*F*	*P*
Water from food	988±253	870±187	934±210	884±185	920±212	1.264	0.293
Water from breakfast	79±35	100±40	82±25	80±26	85±33	1.771	0.160
Water from lunch	463±123	410±99	448±102	449±101	442±107	0.900	0.446
Water from supper	446±130	361±99	404±122	355±107	392±119	2.568	0.055
Water from staple food	413±146	367±114	381±111	352±72	379±115	1.001	0.398
Water from dishes	575±167	503±115	552±137	532±127	541±138	0.965	0.414
24h urine biomarkers							
Volume of urine	939±210	893±187	934±207	830±136	971±231	0.779	0.510
Void number	4±1	4±1	4±1	4±1	5±1	0.279	0.840
Osmolality	973±101	985±104	948±104	997±83	978±98	0.840	0.476
Na (mmol/L)	261±35	247±42	261±33	267±33	259±36	1.015	0.391
K (mmol/L)	43.10±10.99	45.27±13.63	40.68±9.71	51.08±12.83	45.0±12.3	2.523	0.064
Cl (mmol/L)	233±30	213±42	227±31	223±31	224±34	1.165	0.329
Ca (mmol/L)	5.11±2.28	5.18±1.71	4.80±1.56	4.75±1.07	4.97±1.71	0.303	0.823
Phosphorus (mmol/L)	22.24±6.26	26.99±6.69	23.19±5.79	25.42±6.92	24.47±6.58	2.194	0.096

Note: Values are shown as the mean ± standard deviation (SD), with the exception that percentages were shown as n (percentage). Significant differences were found in the urine osmolality, pH and

Mg (mmol/L)	4.38±2.08	4.30±1.74	5.04±2.41	5.08±2.14	4.68±2.08	0.753	0.524
pH	7.0±0.4	6.9±0.5	6.9±0.3	7.1±0.3	7.0±0.4	1.735	0.167
USG	1.023±0.005	1.025±0.004	1.023±0.005	1.022±0.004	1.023±0.004	1.733	0.168
Hydration state (%)							
Optimal hydration	1.7±7.5	0.0±0.0	2.0±6.0	2.6±8.4			
Middle hydration	8.3±14.8	8.0±10.3	12.8±13.5	8.8±13.7		1.630	0.795
Dehydration	90.0±15.7	92.0±10.3	85.2±12.7	88.6±15.2			

USG between males and females (t=-2.368, p=0.020; t=3.125, p=0.003; t=-2.907, p=0.005). But no significant differences were found in the concentrations of Na, K, Cl, Ca, Phosphorus and Mg of urine between males and females (t=0.318, p=0.751; t=0.952, p=0.344; t=-1.575, p=0.120; t=-1.186, p=0.239; t=-0.653, p=0.516; t=0.273, p=0.786). The amounts of water from lunch, supper, dishes, staple food and the water from food were different between males and females (t=5.846, $p<0.001$; t=8.266, $p<0.001$; t=5.451, $p<0.001$; t=7.497, $p<0.001$; t=8.497, $p<0.001$) and the amounts of water from breakfast did not differ significantly (t=1.790, p=0.077).
